# Suppressing Ion Migration in Heterostructure Single Crystals for Highly Sensitive Ultra‐Stable X‐Ray Detection

**DOI:** 10.1002/advs.202507588

**Published:** 2025-06-25

**Authors:** Yu Ma, Wenjing Li, Yi Liu, Wuqian Guo, Haojie Xu, Liwei Tang, Qingshun Fan, Linjie Wei, Junhua Luo, Zhihua Sun

**Affiliations:** ^1^ State Key Laboratory of Functional Crystals and Devices Fujian Institute of Research on the Structure of Matter Chinese Academy of Sciences Fuzhou Fujian 350002 P. R. China; ^2^ Chinese Academy of Sciences University of Chinese Academy of Sciences Beijing 100039 P. R. China

**Keywords:** heterostructure, ion migration, X‐ray detection

## Abstract

Heterostructure single crystals have emerged as a significant functional material system due to their unique properties and potential for novel optoelectronic device applications. Particularly, their distinctive structural characteristics offer promising prospects for suppressing ion migration, which is highly advantageous for X‐ray detection. Herein, by gradually modifying the cyclohexylmethylamine cation into the 4‐aminomethyltetrahydropyran cation, a layered heterostructure single crystal (PbCl_2_)_2_(4‐Aminomethyltetrahydropyran)_2_PbCl_4_ is successfully obtained. It comprises two distinct inorganic frameworks, namely a perovskite layer built from PbCl₆ octahedra and an intergrowth layer consisting of PbCl₂ units, wherein the organic components are firmly anchored to the intergrowth layer via Pb─O bonds, thereby enhancing the crystal stability and effectively suppressing ion migration, as evidenced by high ion migration activation energy (1.64 eV) and extremely low dark current drift (2.86 × 10^−18^ A cm^−1^ V^−1^ s^−1^). X‐ray devices based on the ultra‐stable heterostructure single crystal demonstrate an extraordinary sensitivity of 8453.3 µC Gy^−1^ cm^−2^ (at 40 V), ultra‐low detection limit of 5.2 nGy s^−1^ and superior air stability (≈1 year). Such values make it one of the most outstanding candidates for high‐performance X‐ray detection. This work promotes the development of heterostructure single crystals optoelectronic applications.

## Graphical abstract

This work reports a 2D heterostructure single crystal composed of two distinct inorganic frameworks, of which the ion migration is effectively inhibited by the strong interaction between organic components and inorganic layers. Such merits enable highly sensitive ultra‐stable X‐ray detection performances and highlight the potential of heterostructure single crystals for X‐ray applications.

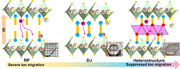

## 1 Introduction

Direct X‐ray detectors can convert X‐ray radiation into electrical signals, which play a significant role in applications such as medical diagnostics, non‐destructive testing, and security screening.^[^ ^1^, ^2^, ^3^, ^4^ ^]^ Traditional X‐ray detectors primarily rely on inorganic semiconductors, including materials such as Si, *α*‐Se, and CdTe.^[^ ^5^, ^6^, ^7^ ^]^ These materials are increasingly unable to meet the rapidly growing demand for X‐ray detection due to their high cost, process complexity, and integration difficulties.^[^ ^8^, ^9^ ^]^ Metal halide perovskites, due to their low cost, ease of fabrication, and advantages such as high mobility‐lifetime product (*µτ*) and strong X‐ray absorption, show great promise as the next generation of X‐ray detectors.^[^ ^10^, ^11^, ^12^ ^]^ For example, 3D (CH_3_NH_3_)PbI_3_ single crystal X‐ray detectors have demonstrated ultra‐high sensitivity (5.2 × 10^6^ µC Gy^−1^ cm^−2^) and extremely low detection limits (0.1 nGy s^−1^), far surpassing traditional semiconductor materials.^[^ ^9^ ^]^ However, these metal halide materials suffer from severe intrinsic ion migration, which can lead to high baseline drift and low signal‐to‐noise ratios (SNR).^[^ ^13^ ^]^ This issue causes instability and degradation of the electronic device performance, thus limiting its long‐term operation and practical application potential.^[^ ^14^ ^]^

Two‐dimensional (2D) heterostructure single crystals that can be self‐assembled in solution have shown significant application potential in the field of optoelectronics due to their unique structural and performance advantages.^[^ ^15^, ^16^, ^17^ ^]^ Compared to traditional homogeneous materials, heterostructure single crystals integrate distinct inorganic structural units through organic components, enabling effective modulation of the physical and chemical properties of materials, while unveiling novel characteristics absent in homogeneous materials.^[^ ^18^, ^19^, ^20^ ^]^ For instance, the heterostructure single crystal (PbBr_2_)_2_(AMTP)_2_PbBr_4_ not only inherits the superior optoelectronic transport properties of metal halide materials but also exhibits in‐plane optical anisotropy;^[^ ^18^ ^]^ the heterostructure single crystal (R/SCPEA)_2_PbI_4_·(R/SCPEA)_2_FAPb_2_I_7_, with its asymmetric multi‐quantum well structure, displays captivating multiple absorption‐emission characteristics.^[^ ^19^ ^]^ Despite considerable progress in the investigation of heterostructure single crystals, research on their role in suppressing ion migration remains limited. Given that heterostructure single crystals possess both high carrier mobility‐lifetime product (*µτ*) and strong X‐ray absorption properties, it is expected that 2D heterostructure single crystals can suppress ion migration and provide great opportunities for high‐performance X‐ray detection.

In this work, we report a 2D heterostructure single crystal, (PbCl_2_)_2_(4‐AMTP)_2_PbCl_4_ (4‐AMTP is 4‐aminomethyltetrahydropyran), achieved by progressively modifying the organic spacer cation. The crystal structure is comprised of two frameworks, namely a perovskite layer built from PbCl₆ octahedra and an intergrowth layer consisting of PbCl₂ units, wherein the organic components are firmly anchored to the intergrowth layer via Pb─O bonds. This enhances crystal stability and suppresses ion migration, as evidenced by a high ion migration activation energy (1.64 eV) and extremely low dark current drift (2.86 × 10^−18^ A cm^−1^ V^−1^ s^−1^). Such a low value is three orders of magnitude lower than that of traditional metal halides. Notably, the X‐ray devices based on the heterostructure single crystal exhibit outstanding performance, including a high sensitivity of 8453.3 µC Gy^−1^ cm^−2^ (at 40 V) and an ultra‐low detection limit of 5.2 nGy s^−1^. Further, the device can be placed in the air for one year without packaging and still maintain a well X‐ray response. This work advances the development of heterostructure single crystals for X‐ray applications.

## 2 Results and Discussion

**Figure 1:**
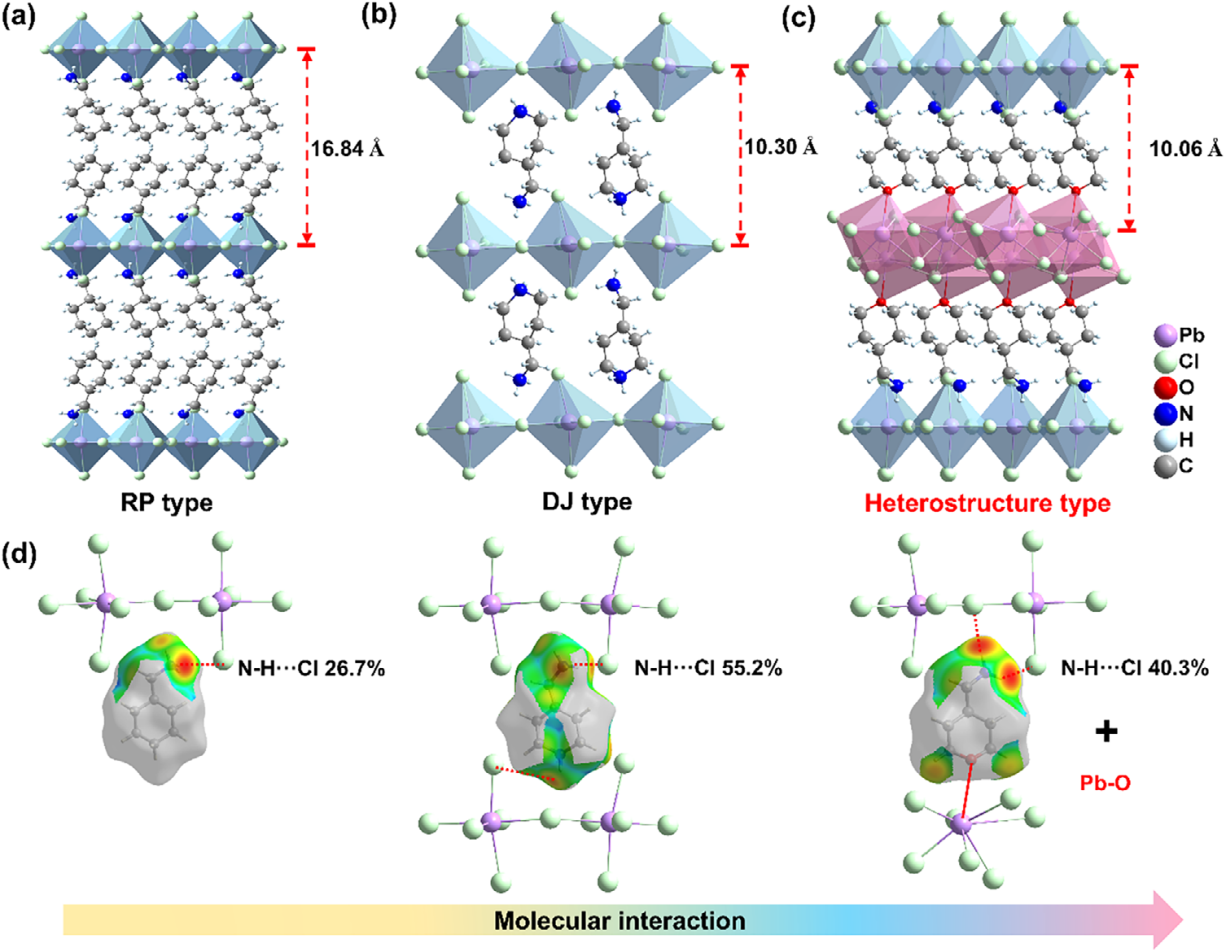
Packing diagram of crystal structures of (CHMA)_2_PbCl_4_ a), (4‐AMPD)PbCl_4_ b), and (PbCl_2_)_2_(4‐AMTP)_2_PbCl_4_ c), respectively. d) Interaction between organic cations and the inorganic framework.

By gradually modifying the organic CHMA cation into the bifunctional 4‐AMTP cation, three representative compounds were synthesized, namely (CHMA)_2_PbCl_4_ (**1**), (4‐AMPD)PbCl_4_ (**2,** 4‐AMPD is 4‐(aminomethyl)piperidine), and (PbCl_2_)_2_(4‐AMTP)_2_PbCl_4_ (**3**). Colorless and transparent block crystals were grown in saturated hydrochloric acid solution by solution cooling method, and the phase purity of the three compounds was confirmed by powder X‐ray diffraction (Figures S1 and S2, Supporting Information). Single crystal X‐ray diffraction reveals that **1** crystallizes in the *Cmc*2_1_ space group,^[^ ^21^ ^]^ exhibiting a typical 2D Ruddlesden‐Popper (RP) type structural feature (**Figure** **1**a). The bilayer organic CHMA cations are orderly arranged between the inorganic layers, forming N─H∙∙∙Cl hydrogen bonds with the inorganic layers through their terminal amine groups. To further regulate the chemical and physical properties of the material, we substituted the C atom on the six‐membered ring of the CHMA cation with N atoms to form the diammonium cation 4‐AMPD through precise chemical modifications. This modification enhances the interaction between the cation and the inorganic layer, therefore compound **2** crystallizes in the *Pca*2_1_ space group, forming a characteristic 2D Dion‐Jacobson (DJ) type perovskite structure (Figure 1b). The single‐layer organic 4‐AMPD cations are connected to the inorganic layers via N─H∙∙∙Cl hydrogen bonds at both terminal amine groups. To explore more complex structural regulation strategies, we further replaced C atoms on the six‐membered ring of the CHMA cation with O atoms to form a bifunctional 4‐AMTP cation. This modification not only introduces additional heteroatomic effects but also enhances the coordination capacity of the cation through the lone pair electrons of the O atom. Finally, compound **3** crystallizes in the *Pnma* space group, forming a distinctive heterostructure with two different sublattices (Figure 1c; Figure S3, Supporting Information). One terminal amine group forms N─H∙∙∙Cl hydrogen bonds with the PbCl_6_ octahedron, while the O atom at the other end bridges with another PbCl_2_ inorganic layer via Pb─O bond. This implies that the organic portion is firmly anchored to the intergrowth layer. A comparison of the three compounds shows that the interlayer distance decreases from 16.84 (**1**) to 10.06 Å (**3**). The shortened interlayer distance enhances the interactions and facilitates charge transport.^[^ ^22^ ^]^ Hirshfeld surface analysis was employed to further investigate the molecular interactions.^[^ ^23^ ^]^ As shown in Figure 1d, the red regions on the Hirshfeld surface represent intermolecular interactions stronger than van der Waals forces (such as hydrogen bonds). The proportions of N─H···Cl interactions on the Hirschfeld surface are 26.7% (**1**, RP‐type), 55.2% (**2**, DJ‐type), and 40.3% (**3**, heterostructure), respectively. However, heterostructure **3** also features strong Pb─O coordination bonds, which firmly anchor the organic cation, far surpassing the hydrogen bond interactions (Figure 1d). This robust interaction will promote lattice rigidity, lock the ion migration pathway,^[^ ^24^ ^]^ and result in superior charge transport, providing the prerequisite conditions for subsequent high‐performance X‐ray detection.

The semiconductor properties are closely related to its band and photoelectric performance, so we performed UV–vis absorption spectroscopy to evaluate the optical bandgaps of the three compounds. As shown in Figure S4 (Supporting Information), the absorption edge of the three compounds is quite similar, with values of 363 (**1**), 360 (**2**), and 358 nm (**3**), respectively. The calculated bandgaps are 3.42 (**1**), 3.44 (**2**), and 3.46 eV (**3**), which are consistent with the results obtained from density functional theory calculations (Figure S5, Supporting Information). We further analyzed the optical properties of **3**, as depicted in Figure S6 (Supporting Information). The valence band maximum is almost entirely composed of Pb 6*s*, Cl 3*p*, and O 2*p* states, while the conduction band minimum is primarily formed by Pb 6*p* and Cl 3*p* states. This clearly indicates that the organic cation and the inorganic framework form an intergrowth layer that is involved in charge transport.

**Figure 2:**
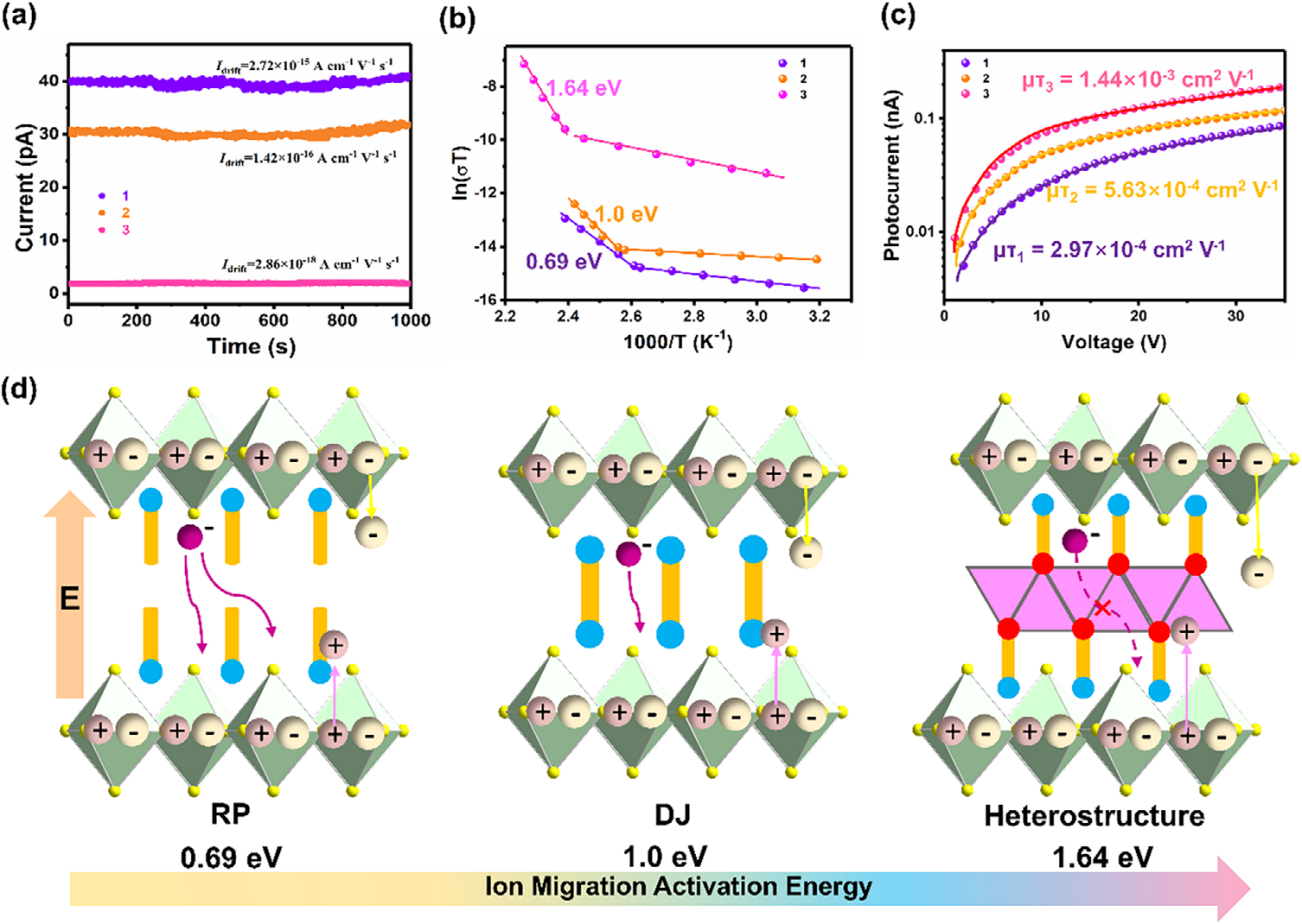
The suppression of ion migration. a) The dark current drift curves of **1**, **2,** and **3**. b) Temperature‐dependent conductivity of **1**, **2,** and **3**. c) The *µτ* of **1**, **2,** and **3**. d) Ion migration scheme in a different single crystal.

To quantitatively characterize ion migration, the dark current drift (*I*_drift_) curves of three compounds were calculated (**Figure** **2**a). The *I*_drift_ of the heterostructure single crystal **3** is 2.86 × 10^−18^ A cm^−1^ V^−1^ s^−1^, which is significantly lower than that of the RP‐type single crystal **1** (2.72 × 10^−15^ A cm^−1^ V^−1^ s^−1^) and the DJ‐type single crystal **2** (1.42 × 10^−16^ A cm^−1^ V^−1^ s^−1^). This value is also much lower than other reported 2D perovskite single crystals,^[^ ^25^, ^26^ ^]^ indicating that the strong coordination bonds in **3** effectively prevent ion migration along the out‐of‐plane direction. To compare the activation energies for ion migration among the three compounds, their temperature‐dependent conductivities were measured. In the low‐temperature region, electron conductivity is the dominant position due to the very low ion concentration. However, ion mobility increases dramatically at high‐temperature, leading to the predominance of ionic conductivity. Figure 2b clearly shows the transition of electronic and ionic conductivities for the three single crystals. According to the Nernst‐Einstein equation:^[^ ^27^ ^]^

(1) $$\sigma \left(T\right)=\frac{\sigma_{0}}{T}\exp\left(\frac{-E_{a}}{k_{B}T}\right)$$

the calculated ion migration activation energies for three compounds are 0.69 eV (**1**), 1.0 eV (**2**), and 1.64 eV (**3**), respectively. The ion migration activation energy of the heterostructure **3** is significantly higher than those of the other two compounds and also exceeds that of other reported 2D metal halides,^[^ ^24^, ^28^ ^]^ further confirming that the intergrowth heterostructure effectively suppresses ion migration. After ion migration is suppressed, the electric field distribution remains uniform and stable. The charge carriers can efficiently and directionally move along the optimal path under the strong electric field, significantly improving the transmission speed and efficiency. This also reduces the trap state concentration and Coulomb scattering, making the carrier transport more coherent.^[^ ^29^, ^30^ ^]^ So with the suppression of ion migration, carrier transport in the out‐of‐plane direction is also enhanced, which is corroborated by the measurements of carrier mobility‐lifetime product (*µτ*) for three compounds (Figure 2c). The *µτ* of **3** is 1.44 × 10^−3^ cm^2^ V^−1^, significantly superior to that of the RP‐type compound **1** (2.97 × 10^−4^ cm^2^ V^−1^) and the DJ‐type compound **2** (5.63 × 10^−4^ cm^2^ V^−1^). This substantial difference in ion migration can be explained by the intergrowth structure of the metal and organic cations shown in Figure 2d. In **1** and **2**, ion migration paths still exist between the organic layers. In contrast, in **3**, the organic cations can form a symbiotic layer with metal ions via coordination bonds, resulting in significant steric hindrance that completely blocks ion migration. All these results highlight the immense potential of compound **3** in X‐ray device applications.

**Figure 3:**
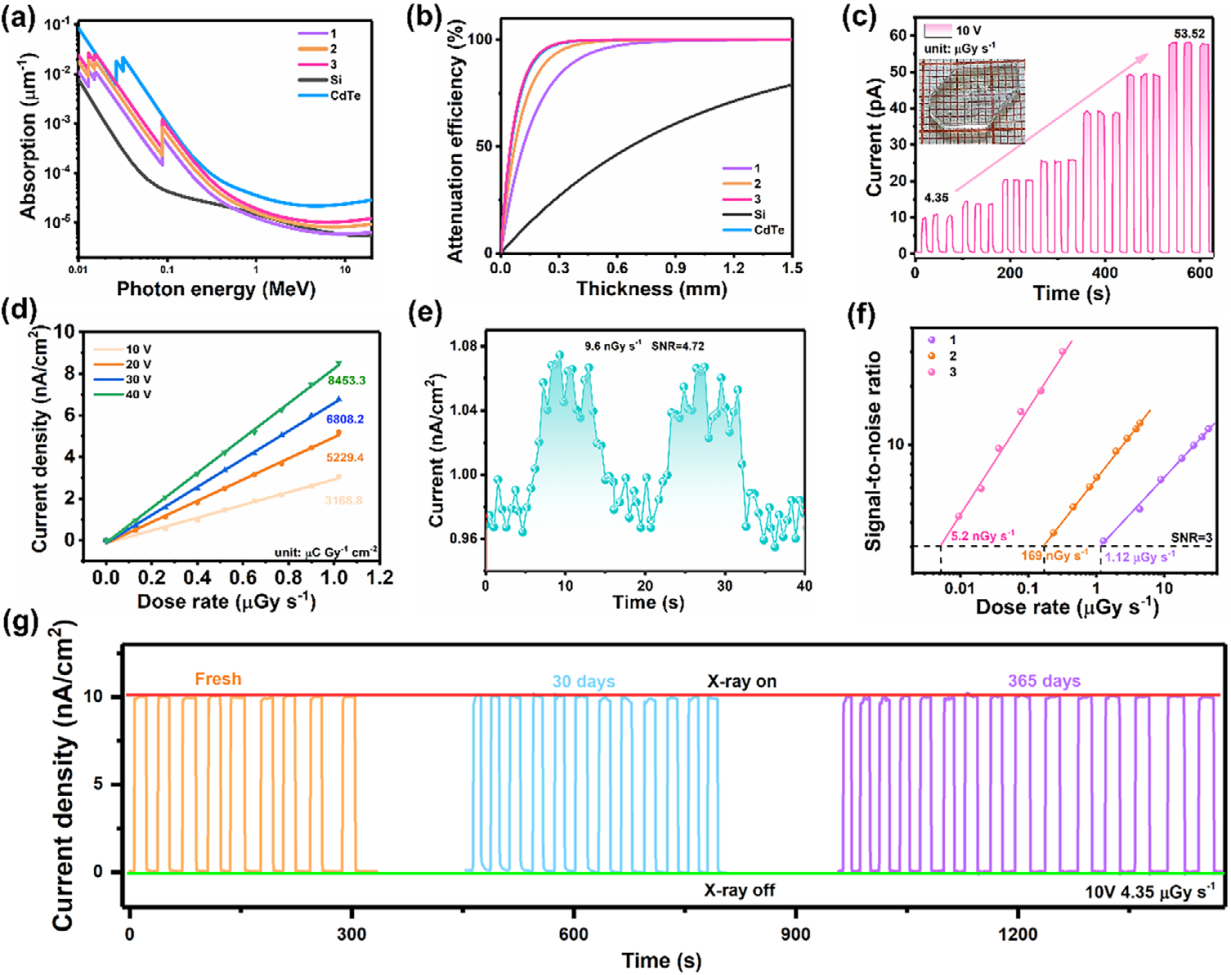
The basic characteristics of X‐ray detection. a) The absorption coefficients of **1**, **2**, **3**, Si, and CdTe. b) The X‐ray attenuation efficiency versus thickness. c) X‐ray response of single crystal device of **3** at different X‐ray dose rates. Inset: centimeter‐scale crystal of heterostructure **3**. d) X‐ray photocurrent density with increased X‐ray dose rates under external voltages rising from 10 to 40 V. e) *I*–*t* curves of device **3** under X‐ray irradiation (9.6 nGy s^−^¹). f) The relationship between SNR and dose rate. g) Long‐term operational stability of single crystal device **3** at the dose rate of 4.35 µGy s^−1^.

Given the unique intergrowth structure and efficient ion migration inhibition of the heterostructure **3**, we anticipate its potential for achieving high‐performance X‐ray detection. We first compared several compounds and conventional semiconductors X‐ray absorption coefficients using the NIST database. Due to its unique heterostructure, compound **3** exhibits a higher density (Table S1, Supporting Information), which results in its X‐ray absorption coefficient being higher than that of compounds **1** and **2**, far exceeding that of the inorganic semiconductor Si (**Figure** **3**a). Specifically, due to the high atomic number of Pb, the X‐ray attenuation efficiency of **3** is markedly higher than that of Si (Figure 3b). This indicates that **3** has a high potential to generate photogenerated carriers under X‐ray irradiation. We fabricated an X‐ray device with a vertical Ag/**3**/Ag architecture, using an Ag‐target X‐ray tube and X‐ray photons to evaluate its detection performance (Figure S7, Supporting Information). As shown in Figure 3c, the single‐crystal device of **3** exhibits a robust switchable X‐ray current response at different X‐ray dose rates, with photocurrent increasing significantly as the radiation dose rate rises, confirming its exceptional X‐ray performance. We then measured the X‐ray response of **3** at 10, 20, 30, and 40 V biases, respectively, and calculated its sensitivity by linearly fitting the current density and dose rate (Figure 3d). As the external voltage increases, charge collection becomes more efficient, and the sensitivity rises from 3168.8 µC Gy^−1^ cm^−2^ (at 10 V) to 8453.3 µC Gy^−1^ cm^−2^ (at 40 V). This value far exceeds many reported metal halide X‐ray devices, such as (*α*‐FPEA)₂PbI₄ (1724.5 µC Gy^−1^ cm^−2^), (3‐MNPA)PbBr_4_ (123 µC Gy^−1^ cm^−2^), and (NPA)₂(EA)₂Pb₃Br₁₀ (225 µC Gy^−1^ cm^−2^).^[^ ^31^, ^32^, ^33^ ^]^

The detection limit is also a critical parameter for evaluating the X‐ray detection performance of devices.^[^ ^34^, ^35^, ^36^ ^]^ We assessed the detection limit of **3** by calculating the signal‐to‐noise ratio (SNR) for different dose rates from the *I–t* curves. As shown in Figure 3e, even at the very low dose rate of 9.6 nGy s^−1^, the device's SNR still reaches 4.72. Further fitting the relationship between SNR and dose rate (Figure 3f), the minimum detection limit of **3** is found to be 5.2 nGy s^−1^, significantly lower than that of **1** (169 nGy s^−1^) and **2** (1.12 µGy s^−1^), and also 106 times lower than the requirements for medical diagnostics (≈5.5 µGy s^−1^).^[^ ^37^ ^]^ Such high sensitivity and low detection limit can be attributed to the contribution of the coordination Pb‐O bond in the structure of **3**, which effectively prevents ion migration. Long‐term stability of the X‐ray device is another important criterion for its practical application. The single‐crystal device **3** exhibits excellent stability at a dose rate of 4.35 µGy s^−1^ for long‐term operation and can remain stable without attenuation after 1 year without encapsulation. After a total X‐ray dose of 1.0 Gy, the photocurrent remains unchanged, indicating the high operational stability of device **3** (Figure S8, Supporting Information). Additionally, thermogravimetric analysis results show that **3** possesses the highest thermal stability, reaching 595 K, which is 72 K higher than **1** (Figure S9, Supporting Information). All these results demonstrate that the heterostructure formed by the Pb─O coordination bond plays a crucial role in the excellent performance of **3**, which holds significant potential for X‐ray device applications.

## 3 Conclusion

In summary, we report a 2D perovskite heterostructure single crystal, (PbCl_2_)_2_(4‐AMTP)_2_PbCl_4_, which contains two distinct inorganic frameworks. As the interactions between organic components and the inorganic sheets increase, crystal stability is improved while ion migration is effectively inhibited, as evidenced by the extremely low dark current drift of 2.86 × 10^−18^ A cm^−1^ V^−1^ s^−1^ and an increased ion migration activation energy of 1.64 eV. Notably, a remarkable sensitivity of 8453.3 µC Gy^−1^ cm^−1^ (at 40 V) and an ultra‐low detection limit of 5.2 nGy s^−1^ were established using X‐ray devices based on the heterostructure single crystal. Furthermore, the device continues to provide stable X‐ray responses with no attenuation even after a year in the air. All these results highlight the great potential of this heterostructure single crystal in the field of X‐ray detection.

## Conflict of Interest

The authors declare no conflict of interest.

## Supporting information



Supporting Information

Supporting Information

## Data Availability

Research data are not shared.
