# Supplementary material for: Suppressing Ion Migration in Heterostructure Single Crystals for Highly Sensitive Ultra‐Stable X‐Ray Detection
Source: Adv Sci (Weinh). 2025 Jun 25;12(36):e07588. doi: 10.1002/advs.202507588 (PMC12462973; doi:10.1002/advs.202507588)
Supplement: Supplementary file 1 — Supporting Information [file ADVS-12-e07588-s002.docx]

Supporting Information

Suppressing Ion Migration in Heterostructure Single Crystals for Highly Sensitive Ultra-Stable X-ray Detection

*Yu Ma,^#^ Wenjing Li,^#^ Yi Liu, Wuqian Guo, Haojie Xu, Liwei Tang, Qingshun Fan, Linjie Wei, Junhua Luo, and Zhihua Sun**

**Experimental Section**.

**Synthesis and crystal growth**: All the chemical reagents and solvents (Adamas-beta^®^) were purchased and used without further purification. During the synthesis process, the stoichiometric ratio of cyclohexylmethylamine, 4-(aminomethyl)piperidine), 4-aminomethyltetrahydropyran and PbCl_2_ were slowly added into the solution of concentrated hydrochloric acid. A clear transparent solution was obtained after the continuous stirring for 60 min at 373 K. Plate-like colorless crystals were obtained by the temperature cooling method after about three weeks, as shown in Figure S1.

**Single-Crystal X-ray Crystallography and Powder X-ray Diffraction.** X-ray diffraction experiments were carried out for **1, 2,** and **3** using a Bruker D8 Quesr/Venture diffractometer with the Mo *Kα* radiation (*λ* = 0.71073 Å). The structures were solved by the direct method and confirmed by the full-matrix least-squares refinements on *F*^2^ using the *SHELXTL* software packing. All non-H atoms were refined anisotropically, and all H atoms were generated by geometrical method and refined by using a “riding” model with *U_iso_* = 1.2 *U_eq_*(C). The above-mentioned structure solution and refinement were conducted in the *Olex*2 software. Crystal data for **1** , **2** and **3** are listed in Table S1. Powder X-ray diffractometry (PXRD) data were recorded on the MiniFlex 600 X-ray diffractometer equipped with a Cu *K*α radiation. Deposited CCDC numbers: 2422891-2422893.

**Characterization.** The UV absorption in solid state was measured at room temperature on a PE Lambda 900 UV-Visible spectrophotometer. Thermogravimetric curves were measured on a STA F4 Jupiter simultaneous thermal analyzer (Netzsch) in a N_2_ atmosphere from room temperature to 800 °C with a heating speed of 10 °C min^−1^. The temperature-dependent conductivities of single crystals were derived from their resistance−temperature curves, which were measured using a high-precision electrometer (6517B, Keithley) by placing samples on a heating−cooling stage (THMS600, Linkam).

**Electronic structure calculations:** Electronic structure calculations of **1, 2,** and **3** was performed by the DFT method by the total-energy code CASTEP, based on the single-crystal structural data. The exchange and correlation effects were treated by Perdew-Burke-Ernzerhof in the generalized gradient approximation. The core-electrons interactions between the ionic cores and the electrons were described by the norm-conserving pseudopotential. The numbers of plane waves included in the basis sets were determined by an energy cutoff 765 eV, and the integration of the Brillouin zone was performed using a Monkhorst-Pack *κ*-point sampling of 6×6×3.

**X-Ray Detection.** The current-voltage (*I*-*V*) traces and current-time (*I*-*t*) curves of **1, 2,** and **3** were recorded by a Keithley 6517B high-precision electrometer. In the optical response measurement, the average interval of the light on/off was 5 seconds. An Amptek Mini-X2 X-ray tube with silver target (maximum power 4 W) was used as the X-ray source. The maximum X-ray photons energy is 50 keV and the peak intensity is at 22 keV. The dose rate of X-ray tube was modulated by changing its tube current and measured by a Radcal Accu-Gold X-ray dosimeter attached with the 10×6-180 ion chamber in an integrating mode. We calculated the absorption coefficient and attenuation efficiency of compounds to X-ray radiation using photon cross-section database. The *μτ* can be calculated using the modified Hecht equation: $I=\frac{I_{0}\mu\tau V}{d^{2}}[1-\exp\left( -\frac{d^{2}}{\mu\tau V} \right)]$, where *I* is the photocurrent, *I_0_* is the saturated photocurrent, *d* is the distance between electrodes, and *V* is the applied bias voltage. The International Union of Pure and Applied Chemistry (IUPAC) defines the dose rate corresponding to an SNR of 3 as the detection limit. The dark current drift is the *I-t* curve of the sample measured at an external bias voltage of 10 V over a long period of time and then calculated according to Equation: *I*_drift_ = (*I_t_*-*I*_0_)/(*E*×A×*t*), where *I_t_* is the current, *I_0_ is* the initial current, *E* and A are the electric field and device area, respectively.

**Figures**


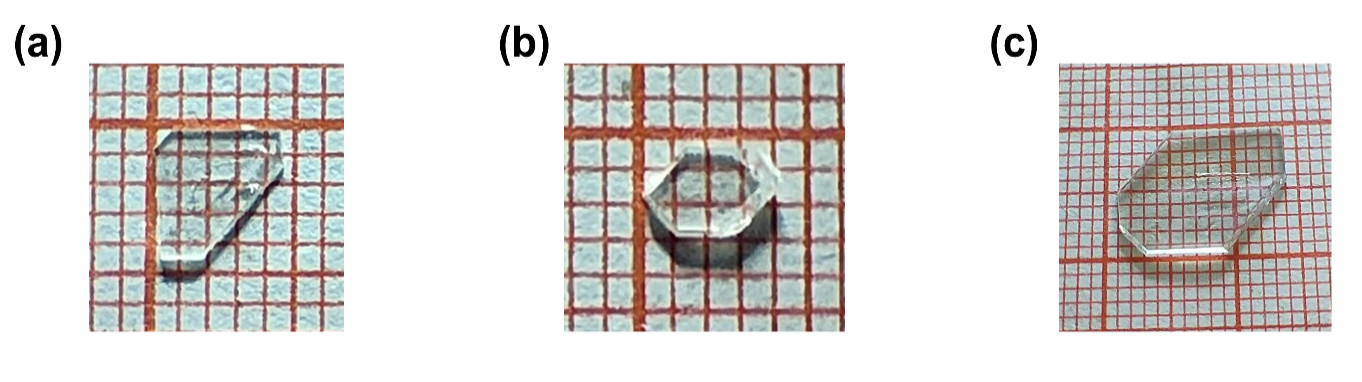


**Figure S1.** Single crystals of **1** (a), **2** (b) and **3** (c) obtained by the temperature-cooling method.


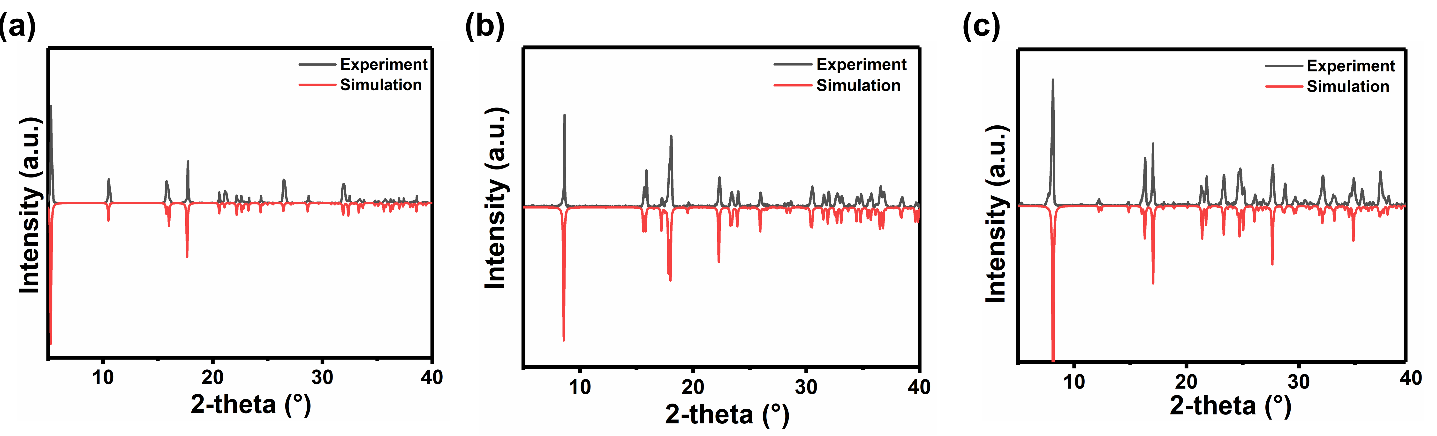


**Figure S2.** Experimental and simulated PXRD patterns for **1** (a)**, 2** (b) and **3** (c) at room temperature.


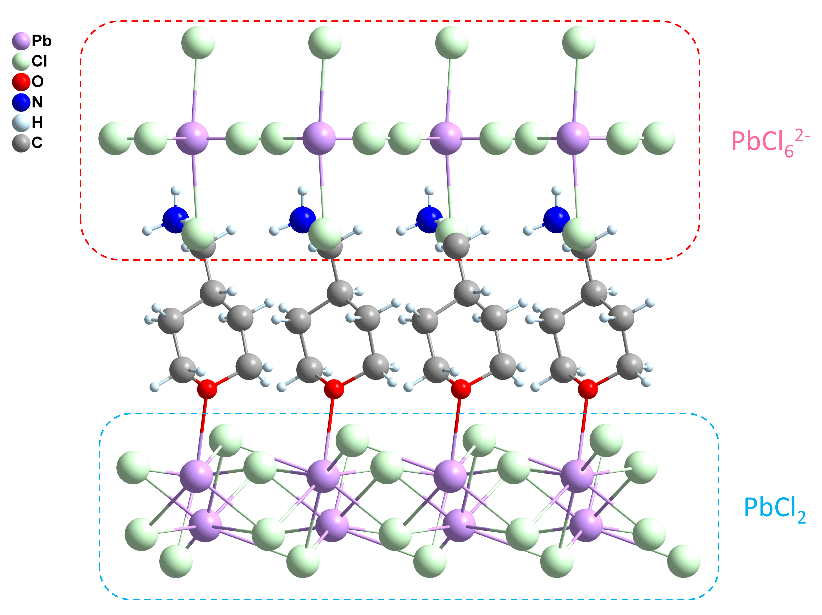


**Figure S3.** Two inorganic frameworks of heterostructural crystal **3**.


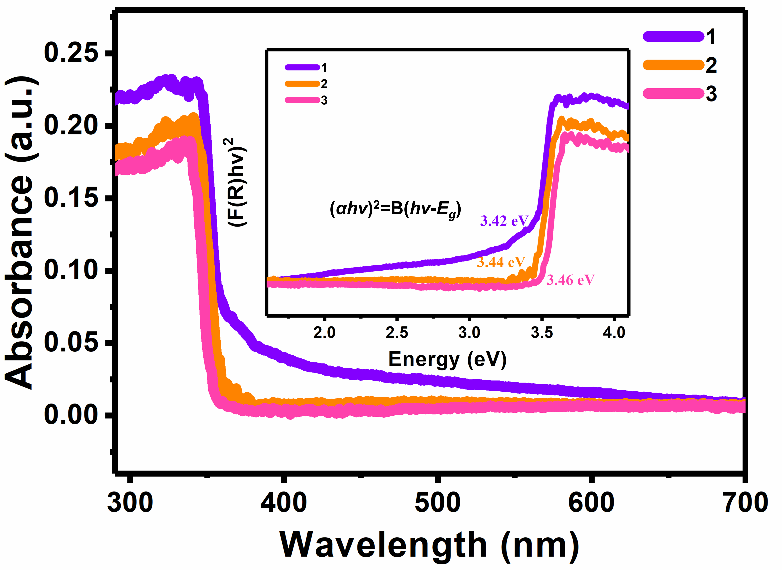


**Figure S4.** The ultraviolet-visible absorption and band gap of **1**, **2** and **3**.


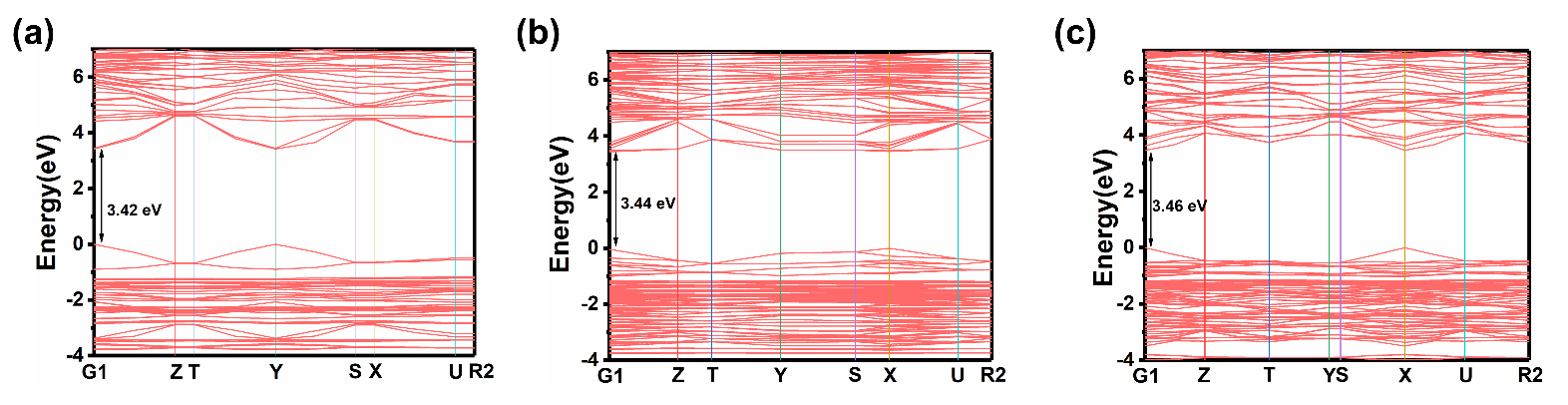


**Figure S5.** The calculated energy band of **1** (a), **2** (b) and **3** (c).


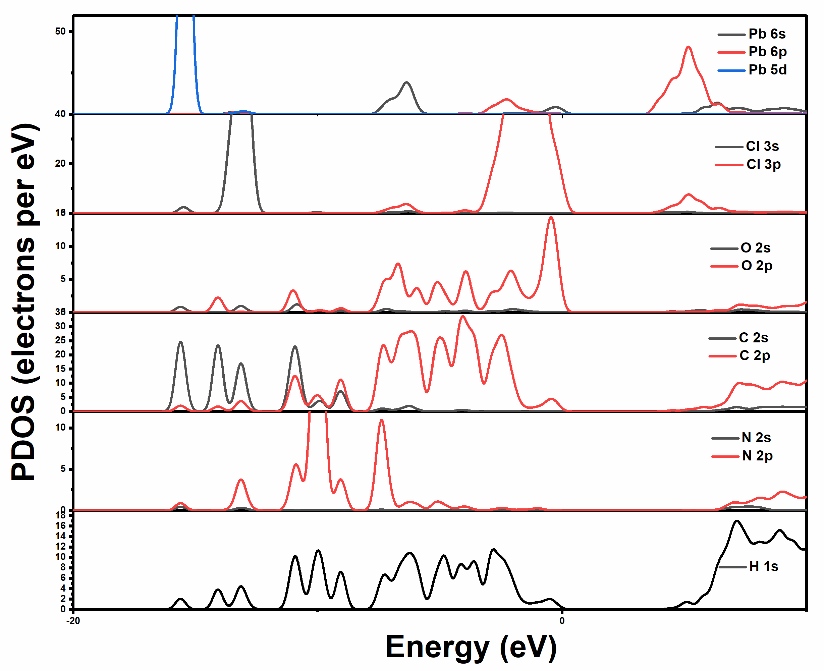


**Figure S6.** The calculated PDOS of **3**.


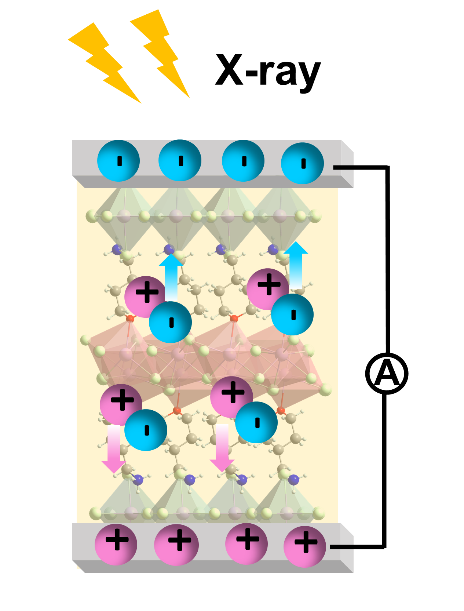


**Figure S7.** The typical schematic diagram of direct-conversion X-ray detector.


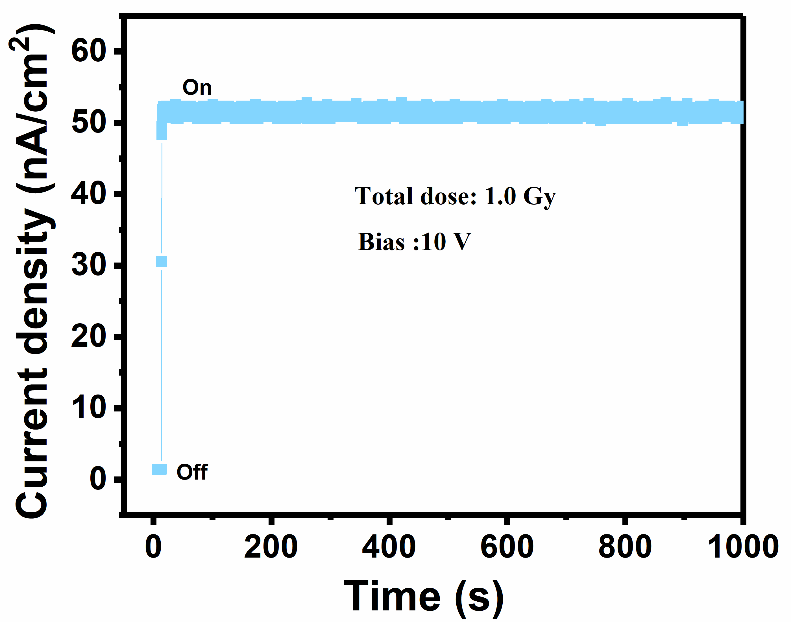


**Figure S8.** Stability of device **3** under prolonged X-ray illumination.


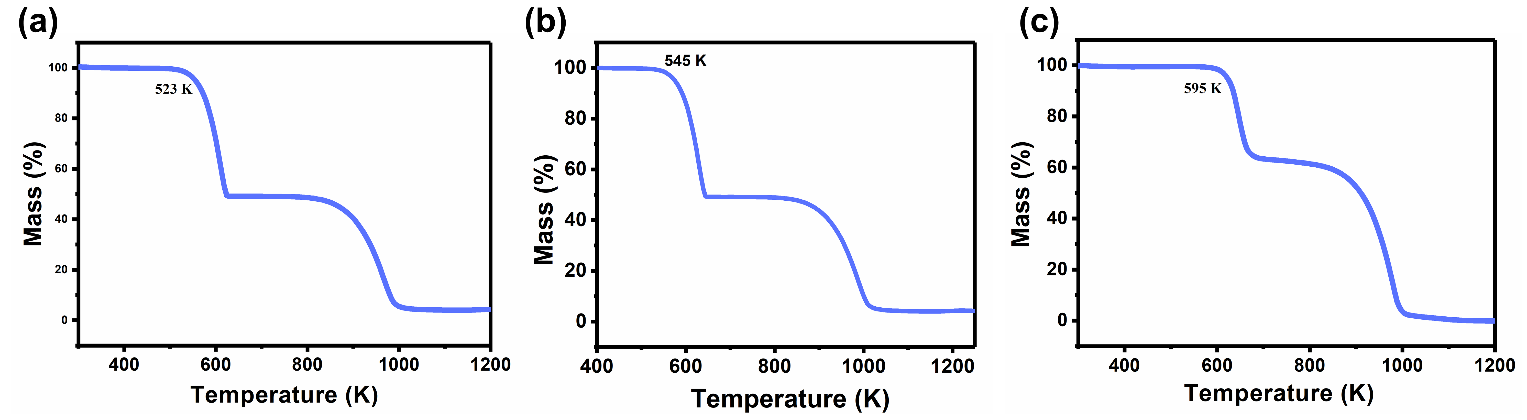


**Figure S9.** The thermogravimetric analysis of **1** (a), **2** (b) and **3** (c).

**Table**

**Table S1.** Crystal data for **1**, **2** and **3**, respectively.

| Empirical formula | C_14_H_32_Cl_4_N_2_Pb (**1**) | C_6_H_16_Cl_4_N_2_Pb (**2**) | C_12_H_28_Cl_8_N_2_O_2_Pb_3_ (**3**) |
| --- | --- | --- | --- |
| Formula weight | 577.40 | 465.20 | 1137.53 |
| Temperature/K | 280.0 | 230.0 | 423.0 |
| Crystal system | orthorhombic | orthorhombic | orthorhombic |
| Space group | *Cmc*2_1_ | *Pca*2_1_ | *Pnma* |
| *a*/Å | 33.689 | 22.6716 | 7.6239 |
| *b*/Å | 8.0079 | 10.2835 | 43.493 |
| *c*/Å | 7.8484 | 11.1841 | 8.1694 |
| *α*/° | 90 | 90 | 90 |
| *β*/° | 90 | 90 | 90 |
| *γ*/° | 90 | 90 | 90 |
| Volume/Å^3^ | 2117.3 | 2607.5 | 2708.8 |
| Z | 4 | 8 | 4 |
| ρ_calc_ (g/cm^3^) | 1.811 | 2.370 | 2.789 |
| μ/mm^‑1^ | 8.470 | 13.724 | 19.401 |
| *F*(000) | 1120.0 | 1728.0 | 2048.0 |
| Radiation | MoKα (λ = 0.71073) | MoKα (λ = 0.71073) | MoKα (λ = 0.71073) |
| 2θ range for data collection /° | 6.248 to 55.048 | 4.35 to 49.996 | 5.074 to 55.006 |
| Index ranges | -43 ≤ *h* ≤ 42,  -10 ≤ *k* ≤ 10, -10 ≤ *l* ≤ 10 | -26 ≤ *h* ≤ 26,  -11 ≤ *k* ≤ 12, -11 ≤ *l* ≤ 13 | -9 ≤ *h* ≤ 9,  -56 ≤ *k* ≤ 55, -9 ≤ *l* ≤ 10 |
| Reflections collected | 6637 | 11484 | 14992 |
| Independent reflections | 2330  [*R*_int_ = 0.0454, *R*_sigma_ = 0.0521] | 3881  [*R*_int_ = 0.1040, *R*_sigma_ = 0.1189] | 3147  [*R*_int_ = 0.0501, *R*_sigma_ = 0.0278] |
| Data/restraints/parameters | 2330/114/101 | 3881/207/232 | 3147/0/128 |
| Goodness-of-fit on *F*^2^ | 1.090 | 1.039 | 1.019 |
| Final *R* indexes [I>=2σ (*I*)] | *R*_1_ = 0.0361, *wR*_2_ = 0.1039 | *R*_1_ = 0.0746, *wR*_2_ = 0.1740 | *R*_1_ = 0.0515, *wR*_2_ = 0.1001 |
| Final *R* indexes [all data] | *R*_1_ = 0.0402, *wR*_2_ = 0.1092 | *R*_1_ = 0.0936, *wR*_2_ = 0.1892 | *R*_1_ = 0.1163, *wR*_2_ = 0.1248 |
